# Cough-Variant Asthma in a Myasthenia Gravis Patient on Treatment With Steroids and Rituximab

**DOI:** 10.7759/cureus.71512

**Published:** 2024-10-15

**Authors:** Shailesh B Meshram, Atulya Anand, Rishi G Orakkan

**Affiliations:** 1 Respiratory Medicine, Dr. D. Y. Patil Medical College, Hospital & Research Centre, Dr. D. Y. Patil Vidyapeeth (Deemed to be University), Pune, IND

**Keywords:** bronchodilator, cough-variant asthma, immunomodulator, myasthenia gravis, rituximab, spirometry, steroids

## Abstract

Muscle weakness that worsens on exercise and improves on resting is the hallmark of myasthenia gravis (MG), an uncommon autoimmune illness. Acetylcholinesterase inhibitors, immunosuppressants, and corticosteroids are commonly used in treatment. Rituximab has been tried in refractory patients. On the contrary, asthma is characterized by a persistent inflammation of the airways and a range of symptoms, including cough-variant asthma (CVA), in which the main symptom is only cough.

We describe the case of a female in her 30s who had a history of MG and had been treated with steroids and rituximab. The patient had an acute dry cough in bouts. Spirometry revealed no obstructive or restrictive pattern, and the chest X-ray was normal. Based on allergy diathesis, a diagnosis of CVA was hypothesized and confirmed clinically by the patient’s reaction to long-acting beta-agonists and inhaled corticosteroids.

This case emphasizes how difficult it can be to diagnose respiratory symptoms in MG patients and how crucial it is to rule out CVA as a differential diagnosis, particularly in patients who have recently started immunomodulatory medication and have an allergy tendency. Fast symptom relief and positive clinical results were achieved by the efficient use of inhalation treatment.

## Introduction

Myasthenia gravis (MG) is an autoimmune disease characterized by muscle weakness that worsens with exertion and improves on rest. The pathogenesis of MG is due to the reduction in the number and activity of acetylcholine receptors at the neuromuscular junction which leads to a decrease in end-plate potential, manifesting as a suboptimal release of acetylcholine during repetitive muscle activity and resulting in muscle weakness [1]. Treatment options for MG include acetylcholinesterase inhibitors, immunosuppressants (azathioprine as an initial choice), corticosteroids, thymectomy, plasmapheresis, and intravenous immunoglobulin [2]. Although most patients, around 80% to 85%, respond well to available treatment options, 15% show poor response to conventional treatment options and require alternative forms of treatment such as rituximab, eculizumab, and cyclophosphamide [3].

In some adults and children, cough may be the only predominant symptom of asthma, and the evidence of airflow limitation may be absent apart from bronchial provocation testing; such a condition is known as cough-variant asthma (CVA). Some patients also develop wheezing and bronchodilator responsiveness in this variant [4].

Both MG and CVA are distinct entities. The coexistence of CVA and MG is a relatively rare occurrence. Exact prevalence rates in terms of percentages can be challenging to determine due to the limited number of reported cases and studies focusing on this specific combination. Although the main effects of MG are on neuromuscular function and the main effects of CVA are on respiratory function, congruent presentation of both conditions is bound to bring uncommon complications and challenges in management. The primary challenge is that the respiratory muscles may be impacted by MG, making diagnosing and managing asthma symptoms more difficult.

Some drugs used to treat asthma, such as beta-agonists, can worsen muscle weakness in MG patients. Oral corticosteroids and biologicals are all part of asthma treatment, which may also be used to treat MG. Thus, the overlap of medications leads to variable manifestations of disease suppression or aggravation, making management of such a case perplexing.

## Case presentation

A female in her 30s presented to the Respiratory Medicine outpatient department (OPD) with a one-week history of cough and throat discomfort. The cough occurred in bouts and was dry in nature. The patient also complained of giddiness during bouts of coughing.

The patient had complaints of muscle weakness for eight years and was diagnosed with MG. The patient was on steroids and biological medications for the same. She did not suffer from any other comorbidities. The patient was treated with intravenous rituximab for MG and received two cycles a month before presentation.

On examination, her vitals were within normal limits, and she was able to maintain a peripheral oxygen saturation of 97%. Respiratory system examination revealed normal vesicular breath sounds on auscultation in bilateral lung fields with no adventitious sounds. Other systemic examinations did not reveal any obvious abnormalities. The patient was admitted to the ward from OPD for further evaluation.

**Figure 1:**
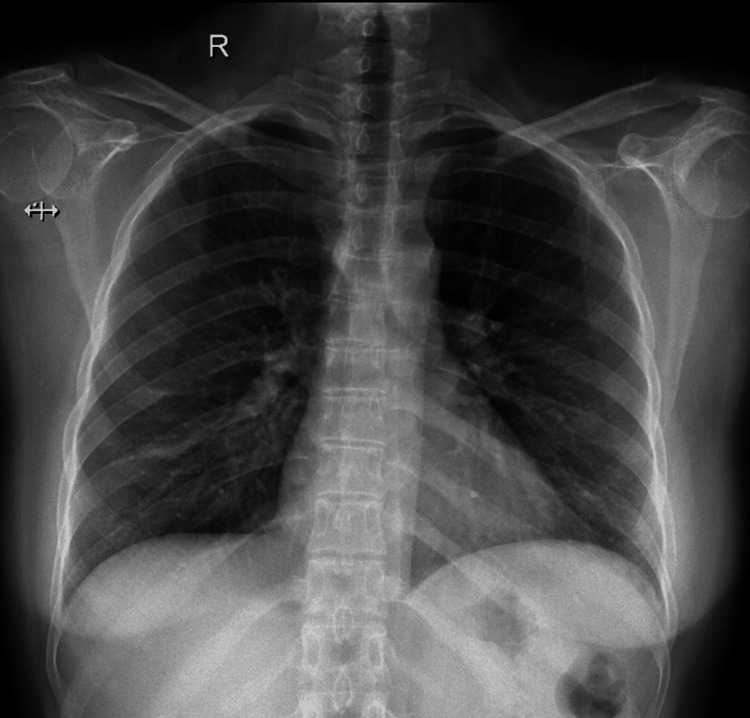
Chest X-ray showing no obvious pleuro-parenchymal abnormality.

The initial presentation of the patient led us to suspect a pathology of infective etiology either due to a bacterial, tubercular, fungal, or viral agent as the patient had received steroids and biologicals. However, the chest X-ray did not reveal any pleuro-parenchymal abnormality (Figure 1), and complete blood counts, including the absolute eosinophil counts, turned out to be normal.

**Figure 2:**
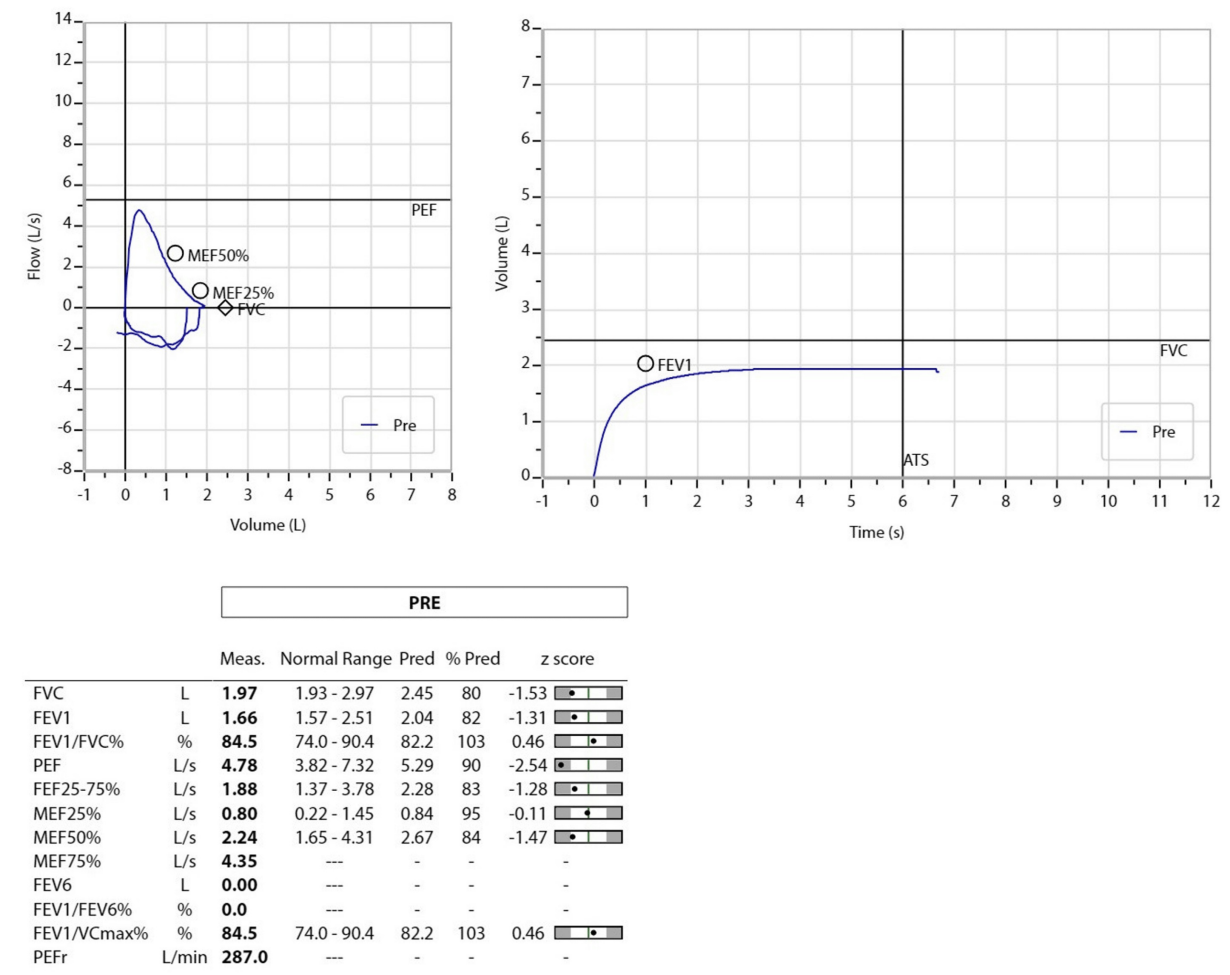
Spirometry showing normal study (FEV1/FV measured at 84.5%, FEV1% predicted at 82 L, and FVC% predicted at 80 L). FEV1: forced expiratory volume in one second is the volume of breath expired with effort in one second; FVC: forced vital capacity is the full amount of air that is expired with effort in a complete breath.

The patient’s functional capability was also assessed with a six-minute walk test that she could not complete because of excessive coughing. Her spirometry revealed a normal study (forced expiratory volume in one second (FEV1)/forced vital capacity (FVC) measured was 84.5%, FEV1% predicted was 82 L, and FVC% predicted was 80 L) with no obstruction or restriction (Figure 2).

**Figure 3:**
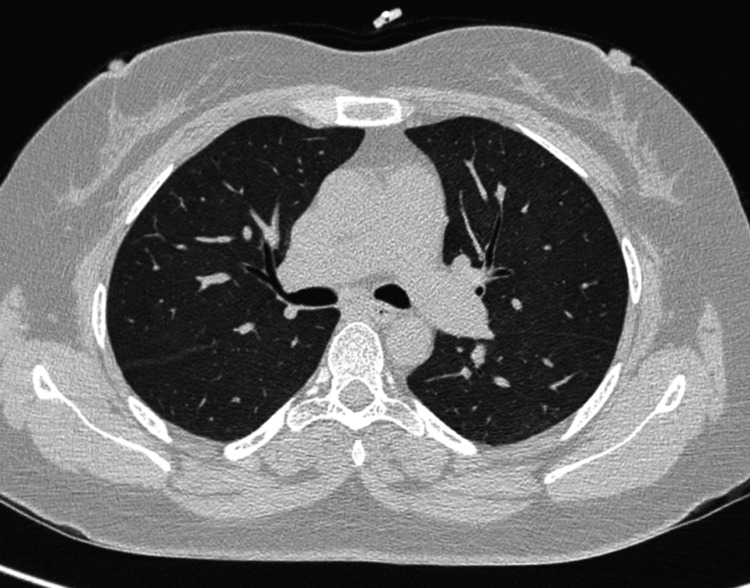
High-resolution CT of the thorax showing no pleuro-parenchymal abnormality.

A transthoracic echocardiography was performed, which showed that the ejection fraction was normal and there were no regional wall motion or valve abnormalities. This was followed up with advanced imaging modalities such as a high-resolution CT of the thorax, which was also suggestive of no pleuro-parenchymal abnormality (Figure 3). These investigations essentially ruled out our suspicions of a pathology that would have been of an infective origin.

This led us to elicit a more comprehensive and detailed clinical history from the patient, during which she gave hints of a few allergic diatheses such as episodic sneezing and rhinorrhea since childhood. She also gave a history of having previously worked as a general help in a woodcarving factory. Our patient was also obese with a body mass index of 30.2 kg/m². Taking these risk factors and additional information into account, we confirmed CVA as our clinical diagnosis.

Based on our diagnosis, she was started on inhaled corticosteroids and long-acting beta-agonists. The patient’s rapid and positive response to bronchodilators within 24 hours supported the diagnosis of CVA. This was proof that our diagnosis of CVA was correct. The patient was prescribed a combination of formoterol and budesonide inhaler at the time of discharge.

The patient was followed up bi-weekly until the complete resolution of symptoms and is on regular follow-up. She has reported a better quality of life and overall confidence since being on regular treatment.

## Discussion

An occult form of asthma in which chronic cough is the only visible symptom is known as CVA [5]. This variant of asthma is in itself a challenge to diagnose because of multiple possible differential diagnoses, such as other variants of asthma, eosinophilic bronchitis, chronic obstructive pulmonary disease, gastroesophageal reflux disease, upper airway cough syndrome or rhinosinusitis, pulmonary fibrosis, and bronchiectasis, all possible pathologies in a patient presenting with chronic cough [6].

MG, a chronic autoimmune disease, is characterized by quick exhaustion and weakening of the voluntary muscles, including the ones involved in respiration. The weakening of the diaphragm and intercostal muscles can eventually lead to respiratory failure [7,8]. Many patients succumbed to pneumonia and respiratory failure before the introduction of acetylcholinesterase [9-11]. Thus, MG patients presenting with respiratory complaints are almost always first suspected of having an infection of the parenchyma and started on empirical antibiotics. Other lung pathologies such as asthma, chronic obstructive pulmonary disease, or emphysema are often missed on first presentation. In most asthmatics, oral steroids are a part of regularly prescribed medications [12]. In patients already on steroids, an acute exacerbation of asthma would not be a major differential. According to the Global Initiative for Asthma guidelines, CVA is generally refractory to treatment with steroids, as seen in our case [13].

MG and asthma share immunopathogenic mechanisms. Previous research has revealed that MG patients have increased serum levels of sCD23. These patients also have overexpression of CD23 in the thymus germinal centers [14]. Substantial improvement in clinical outcomes has been observed following thymectomy which results in decreased serum levels of sCD23. CD23 is known to regulate antigen presentation, IgE production, and B-cell activation. CD23 is sometimes referred to as the low-affinity receptor for IgE (FcεRII). It is thought that IgE plays a role in the symptoms of asthma and other allergies. Dysregulation of CD4 T lymphocytes (Th1, Th2, Th17, and Teg) is believed to be linked to the pathophysiology of MG and asthma. The most significant correlation has been found between HLA genes and the genetic predispositions for MG and asthma, which have been extensively studied. Virus infections play a significant role in initiating and exacerbating asthma attacks as well as acute wheezing episodes in infants. Viral infection and its association with MG have also been suspected. Antiviral mechanisms can be visualized in the thymus of an MG patient. Certain mechanisms have been proposed to explain how viral agents induce MG, including molecular mimicry, cryptic antigens, epitope dissemination, bystander activation, and polyclonal activation [15].

Patients with asthma can have breathlessness as a result of both airway obstruction and respiratory muscle weakness due to MG. If respiratory muscle weakness is not assessed in such patients, they may receive excessive treatment solely for respiratory issues. This can result in underdiagnosis or delay in the diagnosis of critical neuromuscular conditions. Autoimmune conditions such as rheumatoid arthritis, systemic sclerosis, and Sjögren syndrome have been associated with airway hyperresponsiveness, most likely due to structural damage and inflammatory infiltration. Epidemiological studies have also shown a connection between asthma and autoimmunity in type I diabetes [16].

Despite challenges in de-escalating medication regimens, neuromuscular disorders such as MG are often overlooked in patients presenting with acute asthma exacerbations. Both conditions include autoantibodies and have been demonstrated to coexist with other autoimmune disorders such as chronic idiopathic urticaria, inflammatory bowel disease, and autoimmune thyroid disease. MG has been associated with a severe type of non-atopic asthma. Exacerbations of both MG and asthma can lead to fatal consequences. In addition to being a major cause of acute asthma attacks, viruses have also been demonstrated to initiate several autoimmune disorders by molecular mimicry, self-antigen exposure, and tissue destruction. Furthermore, in both conditions, the respiratory muscles are mechanically impaired. It has been demonstrated that MG by itself can manifest as restrictive pulmonary disease. Thus, significant dyspnea may result from the coexistence of MG and asthma. For asthma patients who have difficulty with de-escalation of steroid medication, we should take into account neuromuscular illnesses such as MG [17].

## Conclusions

This case illustrates the diagnostic challenge of managing MG alongside CVA, as both conditions can impact respiratory function and are complicated by overlapping symptoms and treatments. The patient’s positive response to inhaled corticosteroids and beta-agonists confirmed the diagnosis of CVA, highlighting how steroids used for MG may not alleviate asthma symptoms. This underscores the critical need for careful evaluation of respiratory symptoms in MG patients, emphasizing the importance of integrating clinical history with targeted testing to avoid misdiagnosis and ensure effective treatment.
